# Emotional pathways linking relative deprivation experiences to conspiracy beliefs: a mini-meta-analysis

**DOI:** 10.1186/s40359-026-04835-5

**Published:** 2026-06-02

**Authors:** Katharina Abad Borger, Eva Walther

**Affiliations:** Department of Psychology, Social Psychology, Trier University, Trier, Germany

**Keywords:** Anger, Disgust, Anxiety, Relative deprivation, Conspiracy beliefs

## Abstract

**Background:**

Conspiracy beliefs pose a societal challenge, yet the processes through which they emerge remain debated. One pathway to conspiracy beliefs that has received limited attention is the role of emotional responses to relative deprivation (RD). RD — the perception of being unfairly disadvantaged— elicits negative emotions such as anger and disgust, which may in turn foster conspiratorial interpretations.

**Methods:**

Across eight preregistered studies (*N* = 3,306), we experimentally induced direct, individual-level RD using a validated unfair-outcome paradigm and assessed anger, disgust, and (exploratorily) anxiety. All studies measured conspiracy mentality and specific conspiracy beliefs.

**Results:**

We synthesized study-wise effects in a random-effects mini–meta-analysis. Experimentally induced RD reliably increased negative emotional responses (i.e., anger and disgust across all studies). Anger and disgust, but not anxiety were associated with both conspiracy mentality and specific conspiracy beliefs. Mini–meta-analytic mediation analyses showed consistent indirect effects of RD on both conspiracy outcomes through negative emotions. Total effects of RD on conspiracy beliefs, however, were very small and varied in direction across studies, underscoring the importance of emotional mechanisms.

**Conclusions:**

The findings indicate that direct experiences of RD may promote conspiracy beliefs indirectly through heightened anger and disgust. This work highlights results that are consistent with an important role of momentary emotional responses in the emergence of conspiracy beliefs and advances understanding of the affective mechanisms linking RD to conspiratorial thinking.

**Supplementary Information:**

The online version contains supplementary material available at 10.1186/s40359-026-04835-5.

## Background

Conspiracy beliefs surge in times of crisis, when individuals experience uncertainty and perceive their worldviews as destabilized [1]. Conspiracy beliefs are perceived as a threat to democracy, as they undermine trust in political institutions and the legitimacy of elected governments [2, 3]. By portraying secret elites as the true holders of power, conspiracy beliefs fuel distrust in democratic processes, polarize societies through their simplistic narratives of good versus evil [4], and reinforce antisemitism and other exclusionary ideologies [5]. However, the psychological mechanisms underlying conspiracy belief formation remain a subject of debate and intense research.

One recently discussed explanation is that conspiracy beliefs serve as a coping strategy to manage uncertainty by simplifying complex realities into more manageable schemas and narratives [6, 7]. Particularly broad conspiracy narratives, such as QAnon, are often seen as psychological “one-stop shops” that provide apparent satisfying answers to almost all worldly questions [8]. Conspiracy narratives are also adopted because they offer simplified attribution patterns for subjective experiences of injustice [3]. One experience of injustice that may be particularly relevant is relative deprivation (RD).

### RD as a predictor of conspiracy beliefs

RD, the perception of being unfairly disadvantaged compared to others [9, 10], is not merely a belief about disadvantage; it is an evaluative state grounded in appraisals of illegitimacy and external blame. Such perceptions arise from social comparisons with other individuals or groups and reflect subjective evaluations of being unfairly disadvantaged relative to relevant standards. These comparison processes are embedded in everyday social and economic contexts in which individuals encounter and interpret unequal outcomes. (e.g., [9–11]), . Recent work further suggests that such deprivation appraisals may also take a temporal form, reflecting perceived unfair declines in collective status relative to the past (“nostalgic deprivation”; [12]). This conceptual overlap with conspiracy beliefs is striking: Both harbor the desire to blame powerful others for their own disadvantages or for social injustices in a broader sense. Empirically, correlational studies consistently demonstrated that higher RD is associated with stronger conspiracy beliefs across political, economic, and social contexts (e.g., [5, 13–15]), . These studies typically assess RD as a broad, reflective judgment about one’s relative position rather than as a momentary experience. For instance, Ziegele et al. [15] measured perceived economic RD by asking participants to evaluate anticipated changes in their future economic situation and to compare how they are doing relative to others in Germany. Such measures capture global, evaluative perceptions of disadvantage, not momentary unfair events.

Experimental work—though still quite limited—offers complementary evidence, typically by inducing either personal or group-based RD [16, 17]. For example, Gkinopoulos et al. ([16], Study 3) manipulated personal RD by giving participants fabricated feedback about their financial standing relative to other Prolific users. Participants were told that an algorithm had compared their income to that of others. In the *high-RD* condition, they were informed that they would struggle to meet material needs and would need to restrict luxuries; in the *low-RD* condition, they were told they could easily cover needs and occasionally afford luxuries. This induction produced strong differences in perceived personal RD and led participants in the high-RD condition to endorse conspiracy beliefs more strongly.

As another example, Bertin et al. [17] manipulated group-based RD by asking participants to imagine France’s economic future. In the *group-relative-deprivation scenario*, participants imagined France being economically disadvantaged compared to other countries. This increased perceived group deprivation and, particularly among highly nationalistic and narcissistic participants, heightened conspiracy blaming and conspiratorial scapegoating. These studies illustrate that experimentally induced perceptions of unfair disadvantage—whether personal or collective—can increase conspiracy beliefs.

However, important aspects of RD in this context remain understudied. First, to our knowledge, existing work has focused almost exclusively on *chronic* RD—broad, generalized perceptions of unfair disadvantage—rather than RD as a fleeting, situational appraisal. However, it is clear that political behaviour such as voting is influenced by current concerns [18–20]. Second, previous experimental studies typically rely on scenario-based or feedback-based inductions of either group-based RD or chronic personal RD (e.g., imagining national economic decline; receiving comparative socioeconomic feedback [16, 17]. These approaches provide valuable insights but do not capture RD as it can arise in current moments—such as being treated unfairly, losing out to another person, or receiving an inequitable outcome in a situation. Capturing RD at this level of direct, momentary experience, thus, represents a critical next step for understanding how unfairness may translate into conspiratorial beliefs. This gap is meaningful: if conspiracy beliefs emerge in part from how individuals interpret experiences of injustice, event-level RD processes become theoretically crucial. Momentary RD episodes may accumulate and gradually shape broader belief systems, yet these micro-level mechanisms have not been examined in prior work.

### RD and negative emotions: why emotional processes may explain the link

Beyond clarifying *whether* RD predicts conspiracy beliefs, it is equally important to understand *how* this effect unfolds. Identifying the psychological processes activated in the moment of deprivation is essential for explaining why momentary experiences of unfairness might translate into broader conspiratorial worldviews. This shifts the focus from RD as an antecedent to the mechanisms that carry its influence.

RD is not only a cognitive evaluation of unfair disadvantage but also an affective experience [11]. The appraisals central to RD—unfairness, illegitimacy, and external causation—reliably elicit negative emotions, particularly anger and disgust [11, 21–23]. Anger typically arises when individuals interpret disadvantage as unfair and externally caused, particularly in comparison to others who are perceived as better off despite equal input, and is typically directed toward those perceived as responsible, such as specific others, groups, or institutions, motivating efforts to assign blame or restore justice [11, 24–26]. Disgust, in turn, is triggered by perceived moral violations, impurity, or contamination [27, 28] and leads individuals to distance themselves from what they perceive as tainted actors or institutions. These emotional responses map closely onto the logic of conspiracy beliefs, which often portray society as corrupted, immoral, or controlled by malevolent forces. Empirically, anger has been found to heighten conspiratorial interpretations, particularly when events appear intentional or unjust (e.g., [29–32]), and disgust toward political systems or elites predicts stronger endorsement of conspiracy beliefs that depict institutions as corrupt or decayed (e.g., [33, 34]) . Together, these findings suggest that the emotional consequences of RD may be a meaningful pathway through which experiences of unfairness shape conspiratorial worldviews.

Anxiety is also frequently examined in research on conspiracy beliefs and is reliably associated with conspiratorial thinking in contexts of threat and uncertainty (e.g., [35–40]), but also see [41–43] for studies reporting inconsistent effects). However, unlike anger and disgust, anxiety is not theoretically expected to arise directly from RD: the appraisals central to RD—illegitimacy, unfairness, and external blame—do not map onto the uncertainty- and control-related appraisals that typically elicit anxiety [44, 45]. For this reason, we include anxiety in our analysis: it may still correlate with conspiracy beliefs, but it is not expected to serve as a primary emotional response to direct RD.

Taken together, these findings suggest a shared emotional pathway: RD elicits negative emotions, and these emotions—anger and disgust—predict conspiracy beliefs. Yet this theoretically intuitive mechanism remains empirically understudied. Notably, no prior work has examined anger, disgust, and anxiety simultaneously in the context of RD, nor tested whether they jointly help explain why RD predicts conspiracy beliefs.

### Aim of the present research

Although RD has repeatedly been linked to conspiracy beliefs, the existing evidence leaves several important questions open. Most studies rely on correlational designs, and the few experimental approaches that exist have examined either group-based RD or chronic individual RD (e.g., [5, 13, 15–17]), . At the same time, the broader literature—across methodological approaches—has focused almost exclusively on chronic RD, examining stable perceptions of long-term disadvantage. By contrast, direct, individually experienced RD—the immediate sense of being unfairly disadvantaged in a concrete interaction—has, to our knowledge, received no direct empirical attention. As a result, it remains unclear whether such momentary experiences of unfairness causally increase conspiracy beliefs. Understanding these immediate reactions is important because momentary perceptions of unfairness can accumulate and potentially influence broader interpretive tendencies, including conspiracy beliefs.

A second unresolved question concerns why RD relates to conspiracy beliefs. While prior work consistently documents an association between RD and conspiratorial thinking, the psychological processes underlying this link remain underspecified. Separate lines of research show that anger and disgust promote conspiratorial interpretations, and directly experienced RD is known to trigger precisely these emotions (e.g., [30, 31, 33]), . This convergence points to emotional responses as a plausible mechanism connecting RD to conspiracy beliefs, yet this mediating process has not been tested directly to date.

The present research addresses these gaps by synthesizing eight preregistered studies that experimentally induced direct, individual-level RD, assessed anger and disgust (and exploratorily anxiety), and measured conspiracy beliefs. Directly experienced RD was induced with a validated unfair-outcome paradigm (e.g., [46, 47]), , in which participants played a real-stakes coin-win game (the RD game) against an ostensible partner and, in the RD condition, consistently earned substantially less money than the other player. This created a direct, situational experience of unfair disadvantage, simulating a core feature of real-world RD—namely, experiencing disadvantage compared to others despite equal input and without a justified explanation, as commonly observed in everyday social and economic contexts (e.g., unequal pay for comparable work). Across all studies, the same core theoretical process was examined—emotional responses as the link between directly experienced RD and conspiracy beliefs—allowing us to treat the project as a unified research project rather than a set of isolated findings. Accordingly, we conceptualize the process as a sequential model in which directly experienced RD elicits negative emotions that in turn shape conspiracy beliefs. We therefore integrate the evidence into a mini-meta-analysis to obtain a precise and robust estimate of both indirect and direct effects. It should be noted that as only RD was experimentally manipulated, whereas the mediator was not, strong causal inferences at the individual level cannot be drawn from the indirect effects. At the same time, the analytical model provides a theoretically grounded representation of the proposed sequential process.

### Mini-meta-analysis rationale

Over the past 2.5 years, we conducted eight preregistered studies using a shared core experimental design based on the validated RD paradigm. Across all studies, the same preregistered mediation model was examined—RD affecting conspiracy beliefs through negative emotional responses—providing a unified conceptual framework for the entire research project. At the same time, emotional responses were assessed in conceptually comparable ways, although the specific items and the breadth of the emotion measures varied somewhat over time. All studies measured conspiracy beliefs with closely aligned indicators but differed in secondary design features such as additional conditions, moderators, or covariates. This combination of a common theoretical structure, a shared preregistered analytic model, and systematic methodological variation^1^ makes the evidence ideally suited for synthesis in a mini-meta-analysis [50].

Because little prior research had experimentally examined the effects of directly experienced RD on conspiracy beliefs, the earlier studies in the research program were initially planned using more conventional medium-sized effect assumptions. In hindsight, these assumptions were likely too optimistic for situational manipulations of this kind, contributing to relatively small sample sizes and limited power in some of the earlier studies. In addition, several studies were conducted in parallel or embedded in broader laboratory projects with practical recruitment constraints, limiting the possibility of fully adjusting sample sizes iteratively as the research program progressed. At the same time, later studies included substantially larger samples as the likely magnitude of the effects became clearer. Given the small and variable effects observed across individual studies, aggregating all preregistered studies in a mini-meta-analysis provides a more precise and stable estimate of both indirect and direct effects, shifts the emphasis from individual p-values to overall effect sizes, and allows nonsignificant findings to be incorporated transparently rather than treated as anomalies.

A mini-meta-analysis also enables us to address questions that individual studies cannot reliably answer. For example, the direct effect of RD on conspiracy beliefs varied across studies. Although this effect was not the preregistered focus, synthesizing the evidence allows us to determine whether the direct (or total) effect is consistently small, inconsistent, or close to zero.

Finally, integrating the studies into a mini-meta-analysis offers conceptual and practical advantages. Because all studies were guided by the same theoretical model, used a common core design, and targeted the same underlying psychological mechanism, the evidence is best interpreted cumulatively. A mini-meta-analysis, therefore, provides a concise, coherent, and methodologically rigorous way to evaluate the overall pattern across the research project, aligning with current standards for integrated reporting in multi-study projects [50]. Table 1 summarizes the operationalizations, covariates, and sample characteristics of all eight preregistered studies, providing a consolidated overview of the design features underlying the present research.

**Table 1:** Overview of operationalizations, covariates, and sample characteristics across the eight preregistered studies

	Operationalization of RD (Independent variable)	Operationalization of Negative Emotions (Mediator)	Operationalization of Conspiracy Beliefs (Dependent Variable)	Second Manipulation	Covariate & Moderators	Sample Size
Study 1	RD Game (Control, RD, Relative Gratification)	Anger (3 items), Anxiety (1 item)	CMQ (5 items); Specific: generic political conspiracies (3 items) + Ukraine–Russia conspiracies (6 items)	None	Justice Sensitivity; perceived control; revenge motivation; system satisfaction (SPS); positive emotions (happy, satisfied, cheerful); political orientation; political interest; political activity; demographics (age, gender, minority status, nationality, employment)	132 participants (100 women, 30 men, 2 diverse), aged 18 to 33 years (M = 22.74, SD = 2.92), 118 German nationals, 8 dual nationals, and 6 with other nationalities, with most participants being students (116), and a political self-placement on the left–right scale of M = 3.01 (SD = 1.23).
Study 2	RD Game (Control, RD)	Anger (3 items), Anxiety (1 item)	CMQ (5 items); Specific: generic political conspiracies (3 items) + Ukraine–Russia conspiracies (6 items)	None	Justice Sensitivity; perceived control; self-efficacy (ASKU); locus of control (IE-4); revenge motivation; group-based action intentions (ARIS); positive emotions (happy, satisfied, cheerful); political orientation; political interest; political activity; demographics (age, gender, minority status, nationality, employment)	128 participants (98 women, 30 men), aged 18 to 42 years (*M* = 22.28, *SD* = 3.63), 119 German nationals, 5 dual nationals, and 4 with other nationalities, with most participants being students (110), and a political self-placement on the left–right scale of *M* = 2.95 (*SD* = 1.07).
Study 3	RD Game (Control, RD)	Anger (3 items), Anxiety (1 item)	CMQ (5 items); Specific: Ukraine–Russia conspiracies (3 items) + climate conspiracy (1 item) + distrust in experts (1 item) + media/political collusion (1 item) + deep-state conspiracy (1 item) + Great Replacement/ immigration conspiracies (3 items)	None	RD-related experience checks; political statements scale; positive emotions (happy, satisfied, cheerful); political orientation; political interest; political activity; demographics (age, gender, minority status, nationality, employment)	417 participants (258 women, 148 men, 2 diverse, 5 no response), aged 18 to 71 years (*M* = 34.45, *SD* = 13.45), 383 German nationals, 14 dual nationals, and 20 with other nationalities, with most participants being students (116) or employed (201), and a political self-placement on the left–right scale of *M* = 3.40 (*SD* = 1.53).
Study 4	RD Game (Control, RD)	Anger (3 items), Anxiety (1 item)	CMQ (5 items); Specific: generic political conspiracies (3 items) + Ukraine–Russia conspiracies (6 items)	None	Perceived control; belief in science; positive emotions (happy, satisfied, cheerful); political orientation; political interest; political activity; demographics (age, gender, minority status, nationality, employment)	137 participants (114 women, 23 men), aged 18 to 40 years (*M* = 21.35, *SD* = 3.44), 118 German nationals, 10 dual, and 9 with other nationalities, with most participants being students (119), and a political self-placement on the left–right scale of *M* = 3.03 (*SD* = 1.12).
Study 5	RD Game (Control, RD)	Anger (3 items), Anxiety (1 item)	CMQ (5 items); Specific: generic political conspiracies (3 items) + Ukraine–Russia conspiracies (6 items)	None	Need for cognition; perceived control; self-esteem; positive emotions (happy, satisfied, cheerful); political orientation; political interest; political activity; demographics (age, gender, minority status, nationality, employment)	134 participants (91 women, 42 men, 1 diverse), aged 19 to 62 years (*M* = 23.00, *SD* = 4.92), 121 German nationals, 6 dual, and 5 with other nationalities, with most participants being students (107), 14 additionally employed, and 4 not employed, and a political self-placement on the left–right scale of *M* = 3.08 (*SD* = 1.16).
Study 6	RD Game (Control, RD)	Anger (3 items), Disgust (1 item), Anxiety (1 item)	CMQ (5 items); Specific: predominantly left-leaning conspiracies — climate conspiracies (2 items), planned obsolescence (1 item), pharma/alternative-medicine conspiracy (1 item), deep-state/political manipulation (2 items), gender-policy conspiracy (1 item), Israel–Hamas/media conspiracies (2 items), left-wing anti-capitalist conspiracy (1 item)	None	Epistemic trust, mistrust & credulity; positive emotions (happy, satisfied, cheerful); political orientation; political interest; political activity; demographics (age, gender, minority status, nationality, employment)	163 participants (111 women, 48 men, 4 diverse), aged 18 to 76 years (*M* = 24.53, *SD* = 9.86), 145 German nationals, 9 dual, and 9 with other nationalities, with most participants being students (145), and a political self-placement on the left–right scale of *M* = 3.21 (*SD* = 1.34).
Study 7	RD Game (Control, RD)	Anger (3 items), Disgust (1 item), Anxiety (1 item)	CMQ (5 items); Specific: Ukraine–Russia conspiracies (3 items) + climate conspiracy (1 item) + distrust in experts (1 item) + media/political collusion (1 item) + deep-state conspiracy (1 item) + Great Replacement/ immigration conspiracies (3 items)	Emotion manipulation (high vs. low destruction)	Positive emotions (happy, satisfied, cheerful); political orientation; political interest; political activity; demographics (age, gender, minority status, nationality, employment)	1140 participants (866 women, 230 men, 28 diverse, 18 no response), aged 18 to 77 years (*M* = 40.6, *SD* = 12.90), 1081 German nationals, 32 dual, and 27 with other nationalities, with most participants being employed (818), with a political self-placement on the left–right scale of *M* = 3.12 (*SD* = 1.39).
Study 8	RD Game (Control, RD)	Anger (3 DES items), Disgust (3 DES items), Anxiety (3 DES items), Contempt (3 DES items)	CMQ (5 items); Specific: Ukraine–Russia conspiracies (3 items) + climate conspiracy (1 item) + distrust in experts (1 item) + media/political collusion (1 item) + deep-state conspiracy (1 item) + Great Replacement/ immigration conspiracies (3 items) + Trump-related conspiracies (2 items)	Emotion manipulation (high vs. low destruction)	Positive emotions (happiness, 3 items, surprise, 3 items, interest, 3 items); political orientation; political interest; political activity; demographics (age, gender, minority status, nationality, employment)	1055 participants (717 women, 292 men, 29 diverse, 17 no response), aged 18 years to 76 years (*M* = 33.8, *SD* = 14.10), 994 German nationals, 40 dual, and 21 with other nationalities, with most participants being employed (585), with a political self-placement on the left–right scale of *M* = 3.39 (*SD* = 1.58).

### Open practices statement

All data, codebooks (including experimental instructions), and preregistrations of the eight studies are publicly available on the Open Science Framework (https://osf.io/vr5g4/overview). All studies received approval from the university’s ethics committee and were conducted in accordance with the Declaration of Helsinki. Participants were debriefed after study completion.

## Method

### Participants

Across eight pre-registered studies, a total of *N* = 3,306 participants were recruited via the university’s participant management system as well as through social media and targeted online advertisements (e.g., via Meta). Participants received monetary compensation (ranging from €0 to €5.6); in some studies, they additionally received course credit. Sample sizes across the individual studies ranged from *N* = 128 to *N* = 1,140. A detailed overview of demographic characteristics for each study is provided in Table 1.

### Materials and procedure

After providing informed consent, participants received written instructions. Across all studies, the RD game was administered first, followed by the measures of negative emotions and conspiracy beliefs that form the basis of the present mini-meta-analysis. Each study also included additional moderators, conditions, or exploratory measures that are not part of the current analyses. An overview of these study-specific components is provided in Table 1, with full materials and codebooks available in the Online Resources. We used ChatGPT for AI-assisted copy editing and for support in annotating parts of the R script; all analyses, interpretations, and substantive decisions were made by the authors.

### Manipulating negative emotions with the RD game

**Fig. 1:**
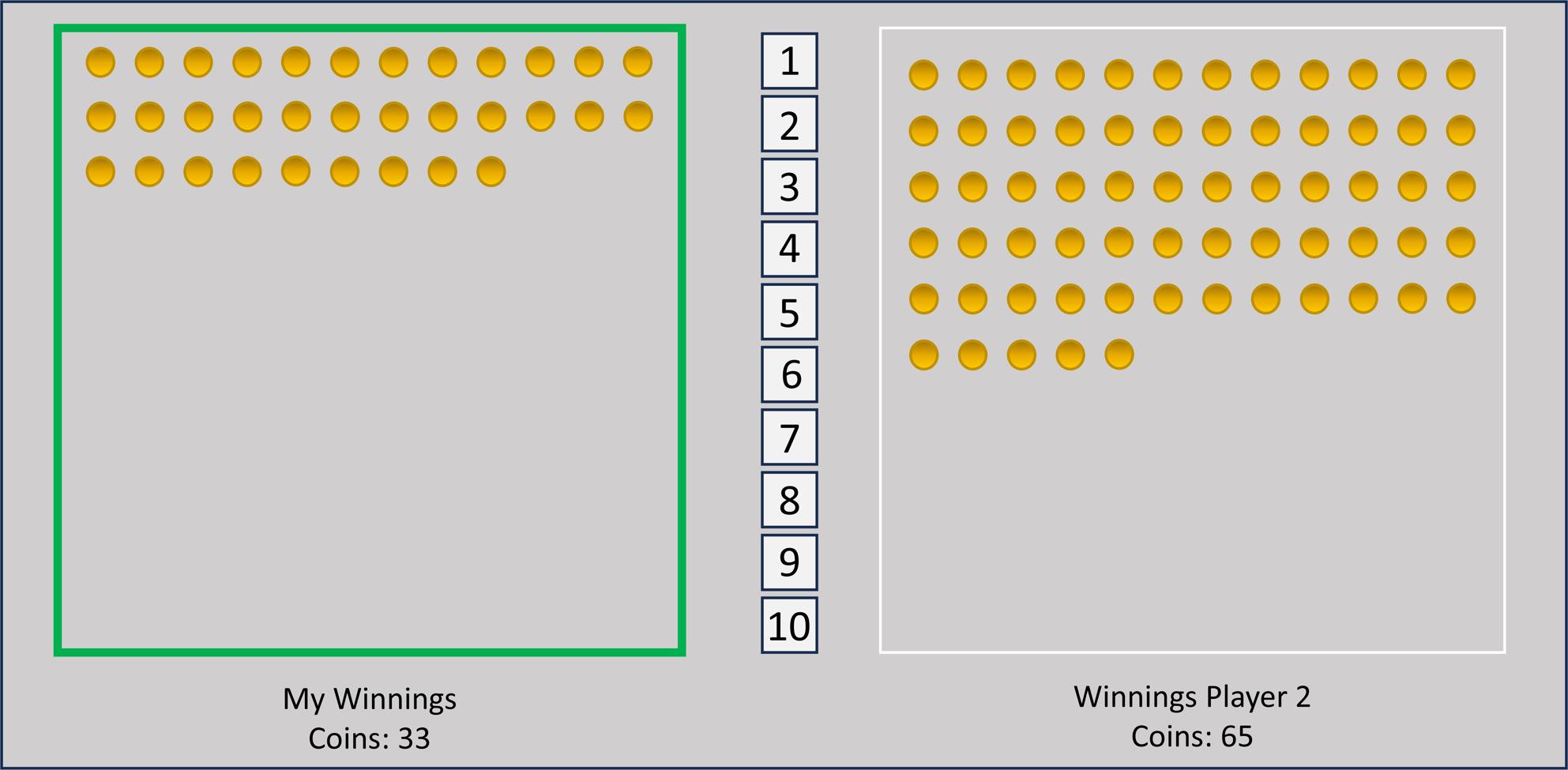
Schematic representation of the game. The display features two coin boxes and ten keys. the green frame of the left coin box indicates that it is the participant´s turn to make a bet

In all eight studies, participants read the following instructions: “You are now playing a game where you can win money. In this game, you play with another person. The money you win will be paid to you in cash (via PayPal) after the game. With the mouse, you can bet on a number (1–10) in the middle of the screen. Either you win this number of coins or not. Your coin box will display the coins you have won. Each coin is worth 5 cents. When it is your turn, your field will light up green. When it is the other person´s turn, their box lights up green.” (see Fig. 1).

In 20 rounds, participants alternated turns with a second player (player 2), who was a game bot. After each turn, feedback appeared at the centre of the screen, indicating which player had won and the number of coins (e.g., “Player 1 won 3!”) or if the bet had been lost (e.g., “Player 1 lost!”). In winning trials, the earned coins were transferred to the player´s coin box, while in the case of a loss, no coins were added. The total accumulated coins were consistently displayed in the coin box. After each trial, the other player had a chance to bet. Participants observed both their own choices and outcomes, as well as those of the opposing player.

In the control condition, both players had equal chances of winning, resulting in approximately similar total payouts at the end. In the RD condition, during the first half of the game, both players also had equal chances of winning to establish the expectation of a fair game (Study 1 included an additional condition; see Table 1). However, starting from the second half, the computer algorithm ensured that the participant´s likelihood of losing and Player 2´s chances of winning increased with each round and, therefore, resulted in a significantly lower payout for the participant compared to Player 2 at the end of the study (see Supplemental Material S4 for specific winning probabilities associated with each key and condition).

### Subjective RD

To test the subjective experience of RD, all eight studies administered a version of the “Personal Perception of Relative Deprivation” scale based on Callan et al. [51] and adapted by Kassab et al. [47]. The scale consisted of six items that assessed on a 7-point Likert scale (1 = *entirely/very fair/very good*, 7 = *not at all/very unfair/very bad*) the participants´ perception of the game and outcome as (un-) fair, of themselves as disadvantaged compared to the other player, and their resulting discontent (e.g., “Compared to the other person: Do you feel disadvantaged?”). Scale reliability was consistently high across studies (ωs > 0.91).

### (Negative) emotions

All eight studies measured negative emotions during the game, using overlapping but partly study-specific emotion items. We measured participants’ negative emotions on a 7-point Likert scale (1 = not at all, 7 = very strong). In Studies 1–7, we assessed anger with three items (“How angry/annoyed/irritated were you during the game?”; all ωs > 0.78) and anxiety with one item (“How anxious were you during the game?”). As distractors, we included several positive emotions (e.g., happy, satisfied, cheerful). In Studies 6–7, we additionally assessed disgust with one item (“How disgusted did you feel during the game?”).

In Study 8, each emotion was assessed with three items adapted from the Differential Emotions Scale [52, 53]. Anger was measured with *irritated*,* annoyed*,* angry*, disgust with *disgusted*,* revolted*,* repulsed*, and anxiety with *fearful*,* scared*,* anxious*. Additional emotions (e.g., positive affect, surprise, contempt, interest) were included as distractors.

Because of the high correlation of anger and disgust (*r* = .48 to *r* = .67), we combined these emotions into a composite of negative emotions (anger only when disgust was not available; mean of anger and disgust when both were available). However, separate analyses for anger and disgust are reported in the Supplemental Material. Anxiety was measured in every study but treated as a separate construct and not included in the focal mediator.

### Conspiracy beliefs

Because previous research distinguished between a general “conspiracy mindset” and the belief in specific conspiracies, all eight studies assessed both constructs separately [2].

### Conspiracy mentality

Conspiracy mentality was assessed in all studies with the Conspiracy Mentality Questionnaire (CMQ; [54]; 5 items, e.g., “I think that many very important things happen in the world, which the public is never informed about”; all ωs > 0.83) on an 11-point scale (0 = certainly not, 10 = sure).

### Specific conspiracies

In addition, participants indicated on an 11-point Likert scale (0 = certainly not, 10 = sure) how much they believed in several specific conspiracy theories. The content of these items varied across studies (e.g., Ukraine war; left-wing conspiracies; Great Replacement; climate change conspiracies). For analytic purposes, these items were always combined into a scale (all ωs > 0.84). A detailed overview of the specific conspiracy items included in each study can be found in the Codebooks in the Online Resources.

## Results

We conducted all analyses using R 4.5.0 (2025-04-11) [55] in RStudio 2025.09.2 + 418 [56]. For the mediation analyses, we used the mediation package [57] with nonparametric bootstrapping (1,000 resamples) to estimate indirect effects and corresponding 95% bias-corrected confidence intervals (CIs). The study-wise mediation effects were then synthesized in a random-effects mini-meta-analysis using the metafor package [58] with restricted maximum likelihood (REML) estimation. Throughout the Results section, we report standardized mean differences (Hedges’ g) for condition differences, partial correlations (rₚ) for associations, and average causal mediation effects (ACMEs) for indirect effects, unless otherwise noted. Note that all eight individual studies were preregistered with the same RD manipulation and mediation hypothesis; early preregistrations also included anxiety as a mediator, but consistent null findings and theoretical considerations led us to discontinue this expectation^2^. For transparency, analyses with anxiety are reported in the Supplemental Material.

### Manipulation check across studies

**Fig. 2:**
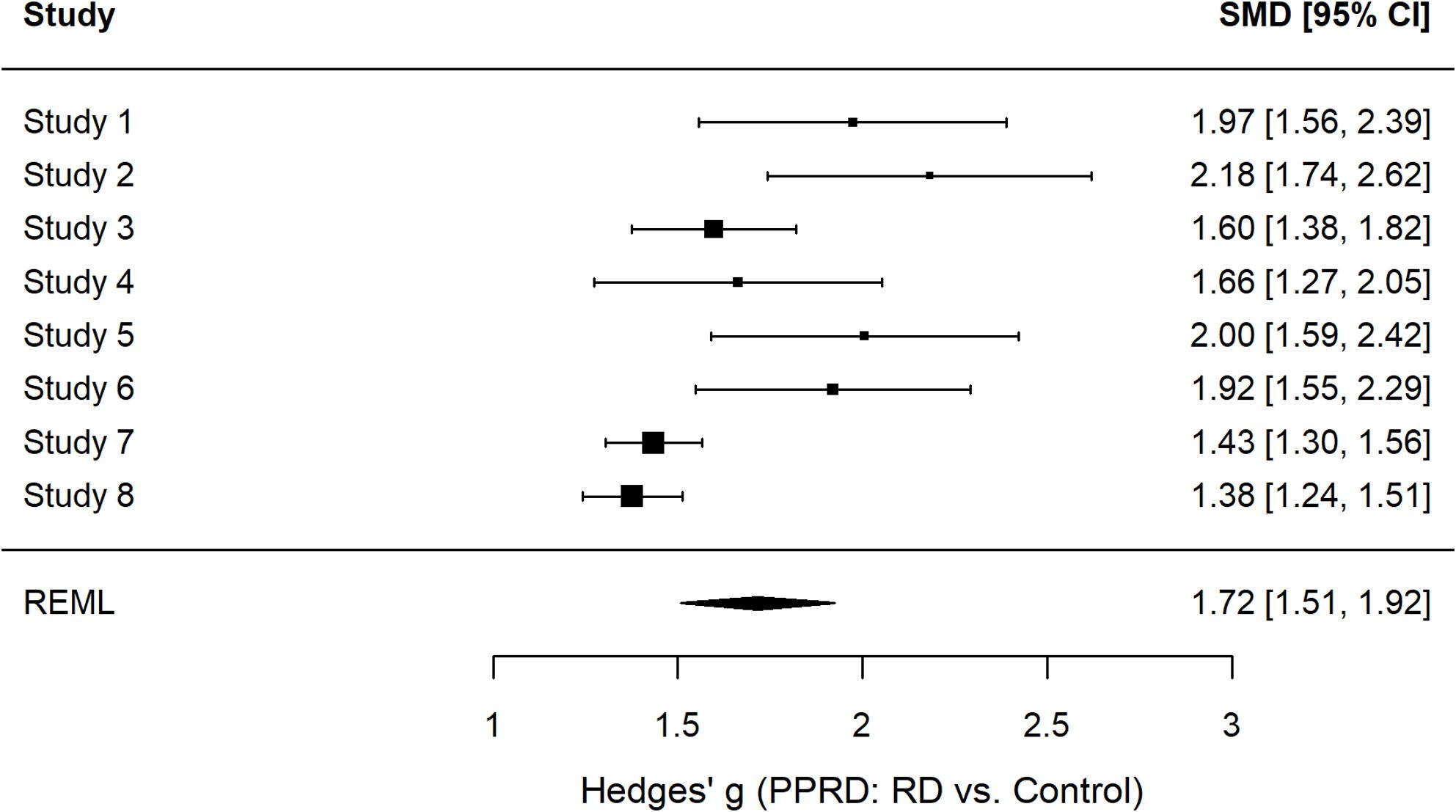
Forest plot of study-wise effect sizes for the manipulation check (Subjective RD). Note. Each point represents the standardized mean difference (Hedges’ g) between the RD and control conditions for one study; horizontal lines indicate 95% confidence intervals. The diamond represents the meta-analytic summary estimate obtained from a random-effects model (REML estimation). Positive values indicate higher perceived unfairness and deprivation in the RD condition compared to the control condition

To test whether the manipulation was successful, we compared participants’ ratings of subjective RD between the RD and control conditions across all studies. A mini-meta-analysis of the eight studies confirmed that participants in the RD condition perceived the RD game as significantly more unfair and reported higher subjective RD than those in the control condition, *g* = 1.71, 95% CI [1.50, 1.93], *p* < .001. Although effect sizes varied across studies (individual *gs* ranged from 1.38 to 2.18), the overall pattern was clear: the RD manipulation reliably elicited substantially stronger perceptions of unfairness and deprivation compared to the control condition (see Fig. 2; see Supplementary Figure S1 for the corresponding funnel plot).

### The link between RD-elicited emotions and conspiracy mentality

#### RD and negative emotions

We first tested whether RD increased negative emotions. A random-effects meta-analysis across the eight preregistered studies showed a robust positive effect, *g* = 0.53, *SE* = 0.09, *z* = 5.87, *p* < .001, 95% CI [0.36, 0.71]. This indicates that participants in the RD condition consistently reported higher levels of negative emotions compared to control conditions. Heterogeneity was substantial, *I²* = 80.11%, *Q*(7) = 37.42, *p* < .001. Taken together, these results suggest that experiences of RD reliably evoke heightened negative emotional responses, although the strength of this effect varied considerably across studies.

### Negative emotions and conspiracy mentality

A random-effects meta-analysis across the eight preregistered studies showed a small but reliable positive association between negative emotions and conspiracy mentality, *rₚ* = 0.13, 95% CI [0.10, 0.17], *p* < .001. Heterogeneity was negligible, *I*² = 0.00%, *Q*(7) = 4.68, *p* = .699, indicating that the effect was highly consistent across studies. Taken together, these results suggest that higher levels of negative emotions are consistently linked to stronger endorsement of conspiracy mentality.

### Indirect association between RD and conspiracy mentality via negative emotions

**Fig. 3:**
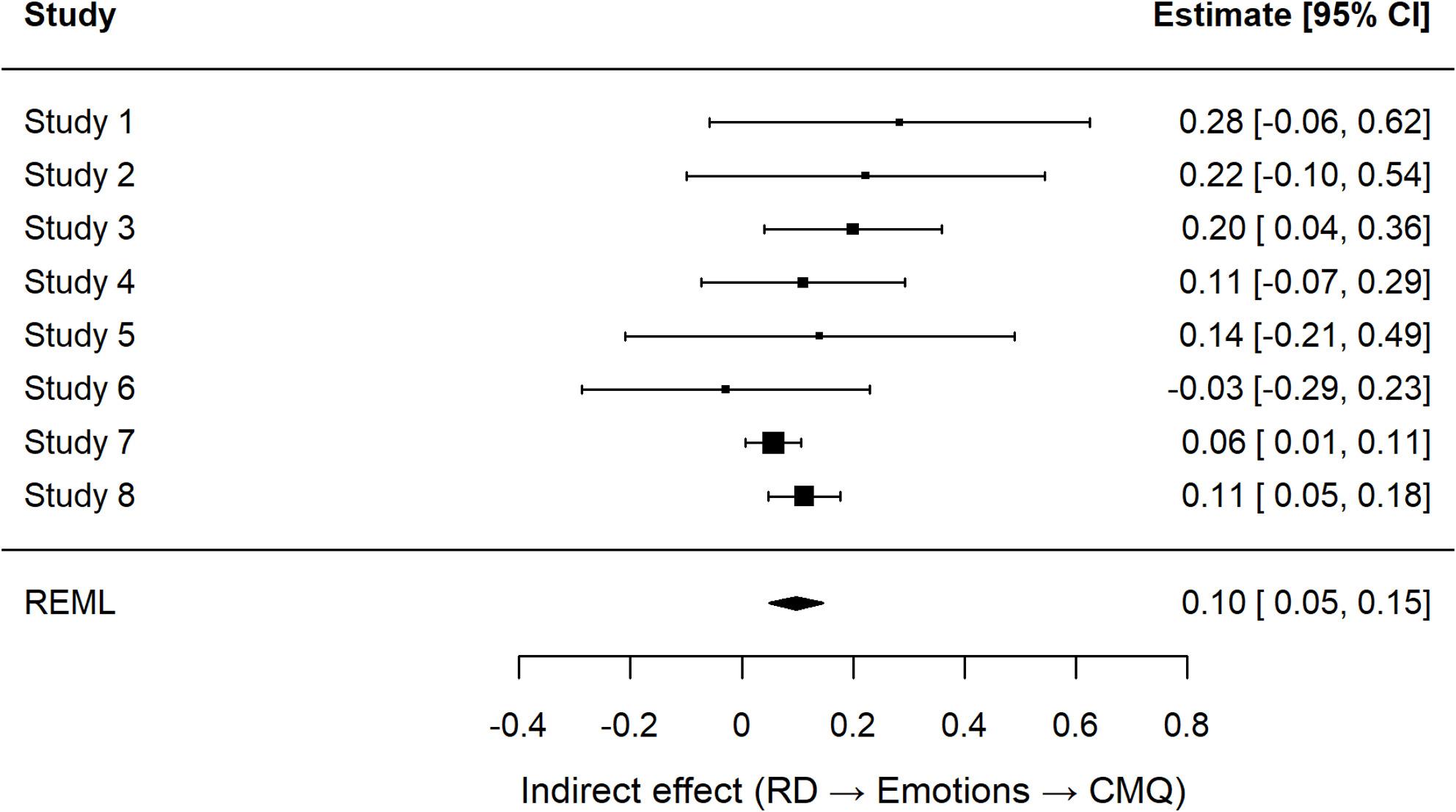
Forest Plot of Study-Wise Indirect Effects (ACMEs) of RD on Conspiracy Mentality via Negative Emotions. Note. Each point represents the study-wise indirect effect, (ACME); horizontal lines indicate 95% confidence intervals. Study-level ACMEs were estimated via nonparametric bootstrapping (1,000 resamples) and synthesized using a random-effects model with REML estimation. The diamond denotes the meta-analytic summary estimate

A random-effects mini–meta-analysis of the study-wise indirect effects (ACME) across studies confirmed a significant indirect effect, ACME = 0.10, *SE* = 0.03, *z* = 3.91, *p* < .001, 95% CI [0.05, 0.15], with low heterogeneity (*I*² = 17.02, *Q*(7) = 6.75, *p* = .455).^3^ Together, these results converge to indicate that the relationship between RD and conspiracy mentality is partly accounted for by heightened negative emotions. Figure 3 displays the forest plot of the study-wise ACMEs with their 95% confidence intervals, illustrating that the indirect effect was consistently positive across studies (see Supplementary Figure S2 and Table S5 for the corresponding funnel plot and study-wise mediation results).

### Total and direct effects

For completeness, we also examined the total and direct effects of RD on conspiracy mentality. The random-effects meta-analysis of the total effect (i.e., without accounting for the mediator) yielded a small and non-significant estimate, *g* = − 0.03, *SE* = 0.03, *z* = − 0.86, *p* = .391, 95% CI [–0.10, 0.04], with negligible heterogeneity, *I*² = 0.00%, *Q*(7) = 2.14, *p* = .952. When controlling for negative emotions, the direct effect of RD on conspiracy mentality was small, negative, and statistically significant, *rₚ =* –0.04, 95% CI [–0.073, –0.004], again with no heterogeneity (*I*² = 0.00%, *Q*(7) = 1.61, *p* = .977). Together, these findings suggest that while the total effect of RD on conspiracy mentality was non-significant and the direct effect was small and negative, the indirect pathway via negative emotions represented the more substantial association between RD and conspiracy mentality^4^.

### The link between RD-elicited negative emotions and specific conspiracy beliefs

#### RD and negative emotions

As reported in the previous section, RD reliably increased negative emotions across all eight studies (*g* = 0.53, *SE* = 0.09, *z* = 5.87, *p* < .001, 95% CI [0.36, 0.71]). Participants in the RD condition consistently reported stronger negative emotional responses than those in the control condition, although the magnitude of this effect varied across studies (*I²* = 80.11%, *Q*(7) = 37.42, *p* < .001). Building on this established effect, we next examined whether these RD-elicited emotions predicted specific conspiracy beliefs.

#### Negative emotions and specific conspiracy beliefs

We next tested whether negative emotions predicted specific conspiracy beliefs. A random-effects meta-analysis across the eight preregistered studies showed a significant positive association, *rₚ* = 0.13, 95% CI [0.10, 0.17], *p* < .001. This indicates that participants reporting higher levels of negative emotions also endorsed stronger conspiracy beliefs. Heterogeneity was negligible, I² = 0.00%, Q(7) = 8.24, *p* = .312. Taken together, these results suggest that negative emotional responses were reliably associated with increased endorsement of conspiracy beliefs, although the strength of this effect varied somewhat across studies.

#### Indirect association between RD and specific conspiracy beliefs via negative emotions

**Fig. 4:**
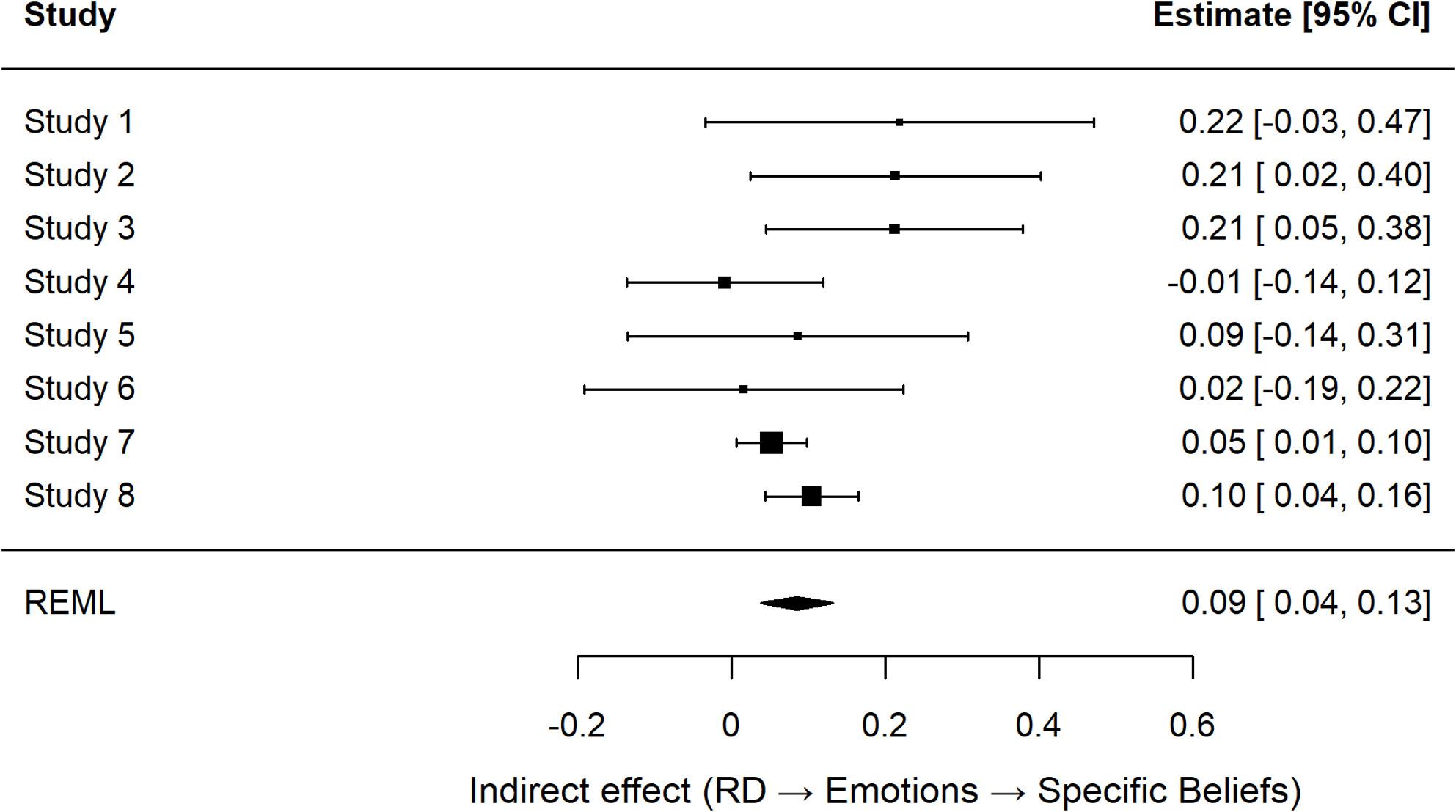
Forest Plot of Study-Wise Indirect Effects (ACMEs) of RD on Specific Conspiracy Beliefs via Negative Emotions. Note. Each point represents the study-wise indirect effect, (ACME); horizontal lines indicate 95% confidence intervals. Study-level indirect effects were estimated via nonparametric bootstrapping (1,000 resamples) and synthesized using a random-effects model with REML estimation. The diamond denotes the meta-analytic summary estimate

A random-effects mini–meta-analysis of the ACME across studies confirmed a significant indirect effect, ACME = 0.09, *SE* = 0.02, *z* = 3.59, *p* < .001, 95% *CI* [0.04, 0.13], with moderate heterogeneity (*I*² = 24.82%, *Q*(7) = 9.68, *p* = .207).^5^ Fig. 4 displays the study-wise indirect effects and corresponding 95% confidence intervals (see Supplementary Figure S3 and Table S6 for the corresponding funnel plot and study-wise mediation results). These findings indicate that negative emotions not only mediate the effect of RD on generalized conspiracy mentality, but also extend to more specific conspiracy beliefs.

### Total and direct effects

To complement the mediation analyses, we also assessed the total and direct effects of RD on specific conspiracy beliefs. The meta-analysis of the total effect (ignoring the mediator) indicated a small and statistically non-significant association, *g* = − 0.02, *SE* = 0.04, *z* = − 0.44, *p* = .661, 95% *CI* [–0.08, 0.05], with virtually no heterogeneity (*I*² = 0.00%, *Q*(7) = 3.06, *p* = .879). When adjusting for negative emotions, the direct effect was small, negative, and did not reach conventional significance thresholds, *r*ₚ = − 0.03, 95% CI [–0.067, 0.002]. Overall, this pattern again suggests that the association between RD and specific conspiracy beliefs was more strongly reflected in the indirect emotional pathway than in the total or residual direct effects of RD.

## General discussion

Although there has been a great deal of research on conspiracy beliefs, the phenomenon remains not well understood. Motivational factors such as RD have been studied in relation to conspiracy beliefs, but their emotional underpinnings have rarely been examined. Moreover, previous work has focused almost entirely on chronic perceptions of long-term disadvantage or on scenario-based manipulations of group or personal RD (e.g., [5, 13–17]). As a result, little is known about how people respond when RD is directly experienced in a concrete unfair interaction, and whether such experiences shape conspiracy beliefs. At the same time, RD is theorized to elicit negative emotions such as anger and disgust (e.g., [23, 59]), which have been both associated with conspiracy beliefs (e.g., [30–32]). Bringing these strands together, the present research examined whether direct RD experiences increase conspiracy beliefs and whether this link is carried by emotional responses.

To address this question, we conducted eight preregistered studies using the same validated RD induction method and the same preregistered mediation model, and synthesized the findings in a mini–meta-analysis. This allowed us to obtain more precise estimates and to overcome the limitations of directly experienced RD and underpowered single studies [50].

The mini-meta-analysis revealed that across eight preregistered studies, the RD game [46, 47] proved to be a robust and reliable tool for inducing direct, individual-level RD experience. Participants in the RD condition consistently perceived the outcome as substantially more unfair and reported markedly higher subjective RD across all studies, demonstrating that the direct experience of RD can be elicited in a controlled experimental setting, thus offering a useful alternative to the scenario- and feedback-based RD inductions commonly used in prior work (e.g., [15–17]) .

Regarding the first research question, the mini–meta-analysis showed that the direct experience of RD did not consistently result in conspiracy beliefs. While the total effects were small and non-significant across outcomes, the direct effects after accounting for negative emotions were small and either non-significant or slightly negative. At first sight, the absence of a positive total effect appears to stand in contrast to previous findings, which have reported positive associations between RD and conspiracy beliefs (e.g., [5, 13–17]). However, the difference between the present and previous findings can be explained when the different RD concepts and measurements are taken into account. The standard definition of RD comprises both a cognitive appraisal of disadvantage component and the affective responses that accompany this appraisal [11]. Prior studies generally assessed RD as a unitary construct—meaning that cognitive and emotional components were not empirically separated. Consequently, previously reported “total effects” of RD on conspiracy beliefs likely reflected this combined cognitive–affective state rather than the cognitive appraisal alone. In our design, these emotional responses were modelled explicitly as a mediator; once they are taken into account, little variance remains for a robust positive direct effect of the cognitive component of direct RD. Rather than contradicting previous findings, our results suggest that emotional responses play a central role in linking RD to conspiracy beliefs. By focusing on momentary RD and experimentally disentangling its components, the present approach provides a more fine-grained understanding of this mechanism. In particular, it allowed us to isolate the (potentially) early processes through which perceived unfairness (RD) translates into conspiracy beliefs.

A further, complementary explanation arises not from the total or direct effects themselves but from the mediation pattern. Because direct experiences of RD elicited negative emotions across all participants, yet only some individuals showed stronger conspiracy beliefs, one possibility is that differences in emotion regulation determine who deals with this emotion by leaning on conspiracy beliefs. Recent work suggests that conspiracy beliefs may function as a response to difficulties in regulating negative affect [60–62]. Applied to our context, this may imply that although the direct experience of RD reliably triggered negative emotions, only individuals who struggled to regulate emotions such as anger or disgust may have been more likely to endorse conspiratorial explanations. This interpretation aligns with the significant indirect effects observed in our mini–meta-analysis and offers a promising direction for future research.

The second research question concerned the psychological processes underlying the RD–conspiracy link. Here, the results converged clearly: Negative emotions were consistently associated with higher conspiracy beliefs, and the meta-analytic indirect effects were significant for both conspiracy mentality and specific conspiracy beliefs. This pattern aligns well with prior work showing that RD reliably elicits negative emotions (e.g., [23, 59]), and that these emotions, in turn, predict conspiracy beliefs (e.g., [29, 31, 33]). The present findings add to this literature by providing empirical support for a plausible pathway: the influence of directly experienced RD on conspiracy beliefs appears to be transmitted, at least in part, through the negative emotional reactions elicited by unfair treatment. Anxiety, examined exploratorily, did not show a comparable pattern (see Supplemental Material for the findings). This is in line with our theoretical reasoning that RD does not necessarily elicit anxiety. It also aligns with prior findings suggesting that the role of anxiety in conspiracy beliefs is not straightforward, as illustrated by research showing associations with both anxious and avoidant attachment styles [63, 64].

A further interesting finding is that not only specific conspiracy beliefs but also conspiracy mentality—a construct commonly described as relatively stable or trait-like (e.g., [2]), —were indirectly associated with directly experienced RD. One possibility is that trait-like beliefs are expressed most strongly when situational cues activate the underlying cognitive schema ([65], see also [66]) for a modern trait activation perspective). An unfair and externally caused disadvantage, as elicited in our RD paradigm, may serve as precisely such a cue, temporarily activating pre-existing conspiratorial tendencies. This would also help explain the small effect sizes: directly experienced RD may not change conspiracy mentality as a relatively stable construct, but may influence how it is expressed in a given situation.

At first glance, this activation perspective might suggest that the role of directly experienced RD is rather limited—that it merely brings to the surface conspiratorial tendencies that already exist and that its impact remains momentary. However, a different interpretation is also conceivable. Although the experimentally induced experience of RD may appear as a simplified representation of the many real-world instances of disadvantage that individuals encounter over time, participants nevertheless responded with pronounced negative emotions such as anger and disgust. This suggests that the experience was subjectively meaningful and psychologically impactful, as such strong emotional reactions would not be expected if the situation lacked perceived relevance. Building on this, a more exploratory idea is that directly experienced RD experiences might not only activate existing conspiratorial schemas but could, over time, also contribute to their formation. This is consistent with research across various psychological domains showing that repeated emotional states can, through processes of reflection and integration, contribute to long-term trait change (e.g., [67–69]). In this view, isolated episodes of directly experienced RD may seem inconsequential, but accumulated across time, they may *s*ediment into more chronic forms of RD, gradually reinforcing suspicious attribution styles and increasing the accessibility of conspiratorial explanations. This possibility remains speculative and cannot be adjudicated with the present data, but future longitudinal or repeated-exposure designs would be well suited to test whether repeated directly experienced RD contributes to the development of more stable conspiratorial worldviews.

Taken together, these findings suggest that the consequences of directly experienced RD unfold primarily through its emotional meaning rather than through a direct shift in conspiratorial thinking. Direct experiences of unfair disadvantage increase conspiracy beliefs only insofar as they evoke negative emotions.

### Strengths, limitations, and avenues for future research

The present project has several methodological strengths—most notably the mini–meta-analytic synthesis of eight preregistered studies using the same validated RD game and preregistered mediation model. The RD game provides a strong and ecologically plausible operationalization of RD. Participants observe others receiving systematically higher outcomes under identical rules, resulting in a clear and externally attributable disadvantage that is experienced firsthand. This structure closely mirrors common real-world situations in which individuals perceive unfair disadvantages, such as unequal pay, unequal returns on comparable contributions, or perceived unfairness in taxation or redistribution [70, 71]. At the same time, this controlled setup necessarily abstracts from the broader socio-cultural and political contexts in which experiences of RD typically occur, which may shape both the nature and intensity of the emotional responses involved.

Future research can build on this paradigm by examining additional forms and contexts of directly experienced RD. For example, extensions could consider situations involving social exclusion, institutional disadvantage, status-based inequality, or more ambiguous responsibility, thereby enriching our understanding of the emotional mechanisms linking RD to conspiracy beliefs across a broader range of real-world experiences.

Regarding future refinement, the differentiation of emotional responses could be a valuable contribution. In the present studies, we focused on anger and disgust as negative emotions, as they are most closely linked to experiences of RD [11, 21–23]. At the same time, this focus reflects a deliberate emphasis on the most theoretically relevant emotional responses to RD and does not preclude the relevance of additional emotions in such experiences. Future research could extend this work by examining a broader range of emotional reactions to RD, as well as the role of further individual difference variables beyond those already considered across the individual studies included in the mini–meta-analysis. For example, contempt has been discussed alongside anger and disgust as part of a broader “hostility triad” underlying moralized judgments [72], and may reflect processes of moral devaluation that could further shape responses to perceived injustice. Emerging work also links conspiracy beliefs to traits such as narcissism and paranoia (e.g., [73–75]), suggesting that additional emotional and motivational states may be relevant in this context.

Moreover, although we aggregated anger and disgust into a composite of negative emotions for theoretical and practical reasons, these emotions serve distinct psychological functions—anger is typically linked to blame and perceived intentionality, whereas disgust is associated with moral contamination and avoidance. At the same time, recent work indicates that individuals higher in conspiracy belief show lower performance-based emotion granularity, meaning that they tend to experience negative affect in a more undifferentiated way and have difficulty distinguishing between closely related negative emotions [76]. From this perspective, our combined measure of anger and disgust may even reflect how participants themselves experience these emotions in moments of unfairness. To determine whether these emotions are indeed experienced as undifferentiated or serve distinct functions in this context, future research could benefit from more fine-grained assessments of emotions, also as anger may vary depending on how the experienced disadvantage is appraised (e.g., as unjust loss or unfair disadvantage relative to others) and at whom responsibility is attributed (e.g., specific others, groups, or institutions).

Regarding limitations, it should be noted that RD was experimentally manipulated, but the mediators were not. As a result, the indirect effects identified here do not permit causal inference at the individual level [77]. This also raises the general possibility—common in mediation-only models—that near-zero total effects alongside positive indirect effects could reflect statistical suppression. In such a pattern, the direct effect would be expected to be consistently negative and of non-trivial size, counteracting a positive indirect effect. In our data, however, this interpretation appears unlikely: Although the pooled direct effect on conspiracy mentality was small and negative, the corresponding total effect remained close to zero, and direct effects were generally small and not consistently negative across outcomes or studies. Importantly, the indirect effect was consistently positive across studies, whereas the direct effects were substantially smaller in magnitude and showed little evidence of a robust or theoretically coherent suppression pattern. In addition, a broad set of covariates (e.g., justice sensitivity, political orientation, perceived control) did not account for either the indirect effects or the near-zero total effects.

In addition, some of the individual studies were likely underpowered when considered in isolation, which may have resulted in imprecise or occasionally inflated estimates, as outlined in the literature on small-study effects and heterogeneity (e.g., [78, 79]). Importantly, however, these concerns primarily arise in the context of selective reporting and unweighted aggregation of imprecise estimates. In the present research program, all preregistered studies—including nonsignificant findings—were included, thereby reducing selective reporting bias, and effect sizes were aggregated using a mini-meta-analytic approach that weights estimates by their precision.

Taken together, the results indicate that emotional responses constitute a meaningful psychological pathway linking RD-elicited emotions to conspiracy beliefs. Future research may strengthen causal inference by experimentally manipulating emotional responses following RD, employing multi-step or properly specified longitudinal designs, or examining emotional trajectories across repeated experiences of unfair treatment.

## Conclusion

This mini–meta-analysis addresses two central questions in conspiracy theory research: whether directly experienced individual RD is associated with conspiracy beliefs, and how such associations unfold through momentary emotional responses. The present findings suggest that directly experienced individual RD does not primarily influence conspiracy beliefs directly, but rather through the negative emotional responses it elicits. In this sense, the controlled experimental paradigm captures a core feature of real-world RD—namely, experiencing unjust disadvantage despite equal input—and thus provides an abstract but socially meaningful model of such situations. At the same time, it offers a basis for extending this line of research to more complex and fully embedded social contexts.

Beyond this, the results show that even momentary unfairness can activate or amplify conspiratorial tendencies, suggesting that situational cues interact with more stable belief systems. Although exploratory, our findings raise a tentative question: whether repeated encounters with momentary unfairness might, over time, play a role in shaping conspiratorial thinking.

Given the societal relevance of conspiracy beliefs, the present findings highlight negative emotions as a central mechanism linking directly experienced RD to conspiracy beliefs. From this perspective, the emotional meaning of RD experiences appears to play a key role in shaping how unfair experiences are interpreted and in creating psychological conditions under which conspiratorial interpretations may emerge.

## Supplementary Information

Supplementary Material 1.

## Data Availability

The datasets generated and analysed during the current study, as well as the analysis code and codebooks (including experimental instructions) are available on the Open Science Framework (OSF; https://osf.io/vr5g4/overview).

## Abbreviations

RD: Relative Deprivation
CMQ: Conspiracy Mentality Questionnaire

## Footnotes

1 The individual studies were originally conducted as part of different subprojects aimed at examining boundary conditions of the same core process (i.e., the link between RD, negative emotions, and conspiracy beliefs). As a result, specific design features (e.g., additional conditions, moderators, mediators, and operationalizations of conspiracy beliefs) were systematically varied across studies, often in relation to specific research questions that are not the focus of the present paper. For example, specific conspiracy belief items were initially based on measures used in prior research within the German context (e.g., [48, 49]), and were subsequently extended to reflect salient societal topics at the time of data collection, that is, issues that were particularly prominent in public discourse in Germany (e.g., geopolitical developments such as the war in Ukraine).

2 Supplementary analyses indicated that anxiety neither emerged as a reliable consequence of RD nor as a consistent predictor of conspiracy mentality or specific conspiracy beliefs. Accordingly, indirect effects via anxiety were uniformly non-significant across studies.

3 Supplementary analyses using the product-of-coefficients approach (a × b; delta method) yielded substantively similar conclusions (see Supplementary Material).

4 Note that direct, indirect, and total effects are derived from separate meta-analytic models with random effects and therefore do not necessarily sum exactly.

5 Supplementary analyses using the product-of-coefficients approach (a × b; delta method) again yielded substantively similar conclusions (see Supplementary Material).
