# Supplementary material for: Emotional pathways linking relative deprivation experiences to conspiracy beliefs: a mini-meta-analysis
Source: BMC Psychol. 2026 Jun 2;14:811. doi: 10.1186/s40359-026-04835-5 (PMC13231626; doi:10.1186/s40359-026-04835-5)
Supplement: Supplementary file 1 — Supplementary Material 1. [file 40359_2026_4835_MOESM1_ESM.docx]

# **Supplemental Materials**

**Anger vs. Disgust as Separate Mediators**

Because anger and disgust were highly correlated across studies (*r* = .48 to *r* = .67), we combined them into a composite of negative emotions in the primary analyses. For transparency, we report the corresponding mediation analyses for anger and disgust separately below.

**Anger as Mediator**

**Conspiracy mentality.** Across the eight preregistered studies, the indirect effect of RD on conspiracy mentality via anger was positive and statistically significant, ACME = 0.096, *SE* = 0.019, *z* = 4.93, *p* < .001, 95% CI [0.058, 0.134]. Heterogeneity was negligible, *I*² = 0.00%, *Q*(7) = 5.34, *p* = .619, indicating that the anger-based indirect effect was highly consistent across studies.

**Specific conspiracy beliefs.** The indirect effect of RD on specific conspiracy beliefs via anger was likewise significant, ACME = 0.078, *SE* = 0.017, *z* = 4.65, *p* < .001, 95% CI [0.045, 0.111], with virtually no heterogeneity (*I*² = 0.17%, *Q*(7) = 7.73, *p* = .357). Thus, RD-related increases in anger consistently accounted for stronger endorsement of specific conspiracy beliefs.

**Disgust as Mediator**

**Conspiracy mentality.** Disgust was available in four of the eight studies. The pooled indirect effect of RD on conspiracy mentality via disgust was positive in direction but not statistically significant, ACME = 0.048, *SE* = 0.033, *z* = 1.46, *p* = .144, 95% CI [–0.016, 0.112]. Heterogeneity was substantial, *I*² = 71.39%, *Q*(3) = 11.58, *p* = .009, suggesting that the magnitude of the disgust-based indirect effect varied considerably across studies.

**Specific conspiracy beliefs.** The indirect effect via disgust was again positive but not statistically significant, ACME = 0.058, *SE* = 0.037, *z* = 1.57, *p* = .117, 95% CI [–0.015, 0.131], with substantial heterogeneity (*I*² = 77.48%, *Q*(3) = 13.69, *p* = .003). As with conspiracy mentality, the disgust-based indirect effects were more variable across the subset of studies in which disgust was assessed.

**The Link Between RD-Elicited Anxiety and Conspiracy Beliefs**

**RD and Anxiety**

We first tested whether RD increased anxiety. A random-effects meta-analysis across the eight preregistered studies showed no reliable effect of RD on anxiety, *g* = −0.03, *SE* = 0.06, *z* = −0.52, *p* = .604, 95% CI [−0.16, 0.09]. Although effect sizes varied across studies, the overall pattern indicated that participants in the RD condition did not report higher anxiety than those in the control condition. Heterogeneity was moderate, *I*² = 58.94%, *Q*(7) = 17.27, *p* = .016. Taken together, these results suggest that, in contrast to other negative emotional responses, anxiety was not reliably elicited by experiences of RD.

**Anxiety and Conspiracy Mentality**

Next, we examined whether anxiety was associated with conspiracy mentality. A random-effects meta-analysis across the eight preregistered studies showed a small positive association between anxiety and conspiracy mentality, *rₚ* = .07, 95% CI [.00, .13], *p* = .042. Heterogeneity was substantial, *I*² = 62.83%, *Q*(7) = 15.93, *p* = .026, indicating considerable variability in the strength and direction of this association across studies. Overall, these findings suggest that anxiety is rather weakly and inconsistently related to conspiracy mentality.

**Indirect Association Between RD and Conspiracy Mentality via Anxiety**

We quantified the indirect effect of RD on conspiracy mentality via anxiety using two complementary approaches. First, applying the product-of-coefficients approach (a × b) based on the meta-analytic estimates above yielded a non-significant indirect effect, *ab* = −0.004, 95% CI [−0.020, 0.012] (delta-method). Second, a random-effects mini–meta-analysis of the study-wise indirect effects (ACME) across studies also showed no evidence for a reliable indirect effect, ACME = −0.01, SE = 0.01, *z* = −0.60, *p* = .550, 95% CI [−0.04, 0.02], with low heterogeneity (*I*² = 22.77%, *Q*(7) = 8.02, *p* = .331). Together, these results indicate that anxiety does not mediate the relationship between RD and conspiracy mentality.

**Total and Direct Effects**

For completeness, we also examined the total effect of RD on conspiracy mentality in the anxiety models. A random-effects meta-analysis across the eight preregistered studies yielded a small and non-significant effect, *g* = –0.03, SE = 0.03, z = –0.86, *p* = .391, 95% CI [–0.10, 0.04], with negligible heterogeneity, *I*² = 0.00%, *Q*(7) = 2.13, *p* = .952. Thus, RD was not directly associated with conspiracy mentality when anxiety was not taken into account.

**The Link Between RD-Elicited Anxiety and Specific Conspiracy Beliefs**

**RD and Anxiety**

As reported above, RD did not reliably increase anxiety across the eight studies (*g* = −0.03, *SE* = 0.06, *z* = −0.52, *p* = .604, 95% CI [−0.16, 0.09]). Participants in the RD condition did not consistently report higher anxiety than those in the control condition, despite moderate heterogeneity across studies (*I*² = 58.94%, *Q*(7) = 17.27, *p* = .016). Building on this result, we next examined whether anxiety predicted endorsement of specific conspiracy beliefs.

**Anxiety and Specific Conspiracy Beliefs**

We tested whether anxiety was associated with specific conspiracy beliefs while controlling for experimental condition. A random-effects meta-analysis across the eight preregistered studies yielded no reliable partial association, *rₚ* = .05, 95% CI [−.020, .120], *p* = .162. Heterogeneity was substantial, *I*² = 65.92%, *Q*(7) = 18.23, *p* = .011, indicating that the relationship between anxiety and specific conspiracy beliefs varied markedly across studies.

**Indirect Association Between RD and Specific Conspiracy Beliefs via Anxiety**

We again quantified the indirect effect using two complementary approaches. Using the product-of-coefficients approach (a × b), the indirect effect of RD on specific conspiracy beliefs via anxiety was not significant, *ab* = −0.002, 95% CI [−0.013, 0.009], *p* = .631.

In addition, a random-effects mini–meta-analysis of study-wise indirect effects (ACME) confirmed the absence of a reliable indirect effect, ACME = −0.01, SE = 0.01, *z* = −1.22, *p* = .223, 95% CI [−0.03, 0.01], with negligible heterogeneity (*I*² = 1.19%, *Q*(7) = 4.63, *p* = .705).

**Total and Direct Effects**

We further examined the total effect of RD on specific conspiracy beliefs, again without accounting for anxiety. The random-effects meta-analysis revealed a small and non-significant effect, *g* = –0.01, SE = 0.03, z = –0.33, *p* = .74, 95% CI [–0.08, 0.06], with no evidence of heterogeneity, *I*² = 0.00%, *Q*(7) = 2.91, *p* = .89. This indicates that RD did not exert a meaningful total effect on specific conspiracy beliefs.

**Summary**

In contrast to negative emotions such as anger and disgust, anxiety neither emerged as a reliable emotional consequence of RD nor as a consistent predictor of conspiracy beliefs. Across both generalized conspiracy mentality and specific conspiracy beliefs, indirect effects via anxiety were uniformly non-significant. These supplementary analyses therefore underscore the specificity of the emotional mechanism identified in the main analyses and suggest that RD-related conspiracy endorsement is not driven by anxiety.

**Supplementary Product-of-Coefficients Mediation Analyses**

**Indirect Effect on Conspiracy Mentality (a × b).** Using the product-of-coefficients approach (a × b) based on the meta-analytic estimates above, we obtained a standardized indirect effect of ab = 0.07, 95% *CI* [0.04, 0.10] (delta-method).

**Indirect Effect on Specific Conspiracy Beliefs (a × b).** Using the product-of-coefficients approach (a × b), the standardized indirect effect on specific conspiracy beliefs was ab = 0.09, *SE* = 0.02, *z* = 4.19, *p* < .001, 95% *CI* [0.05, 0.13].

**Manipulation Check (PPRD)**


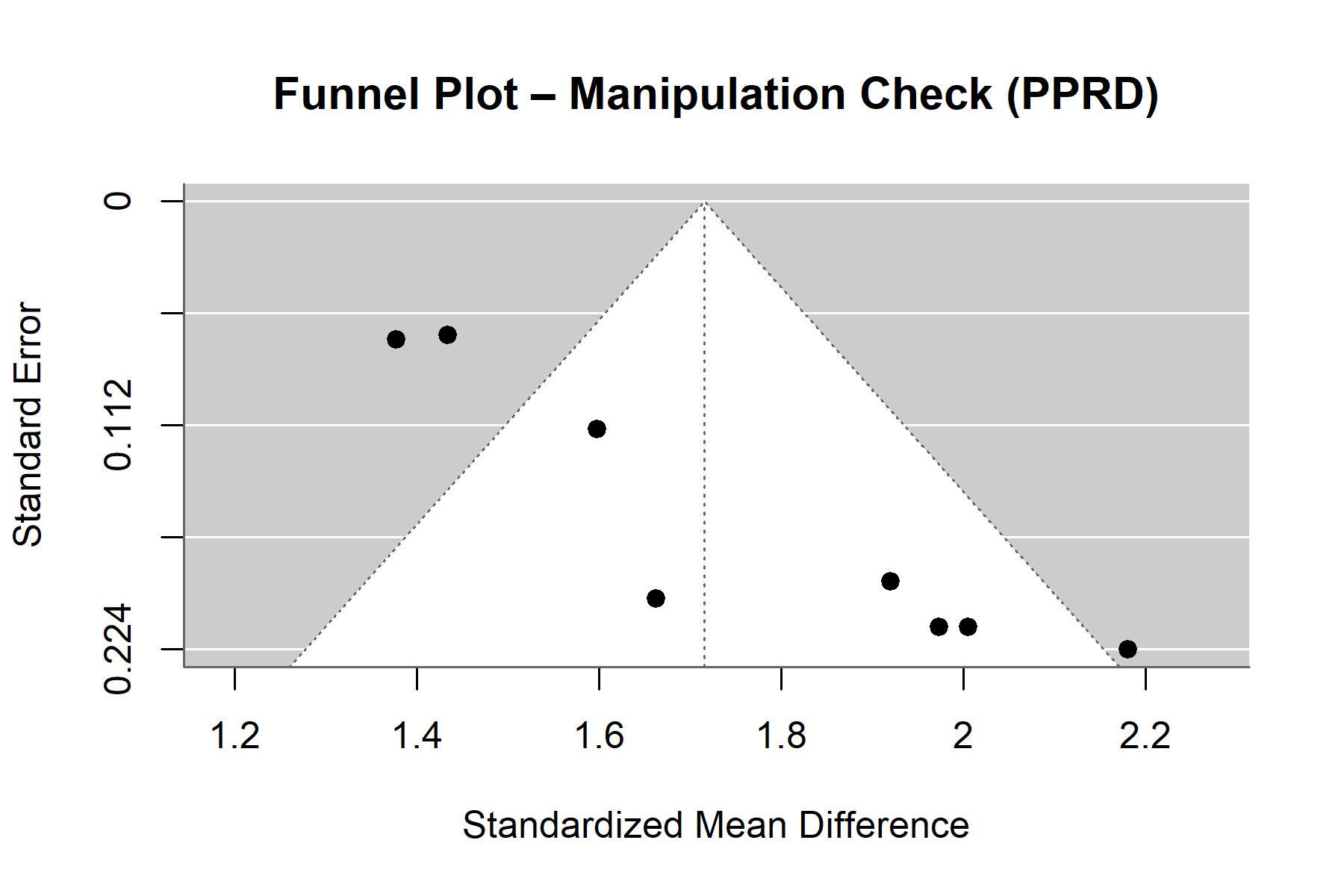
**Figure S1**
*Funnel Plot for the Manipulation Check (Subjective RD).*

*Note.* Each point represents the effect size (Hedges’ *g*) for one study. The vertical line indicates the pooled estimate from the random-effects model. The plot is used to assess potential asymmetry in the distribution of effect sizes

**Indirect Effect (RD → Emotions → CMQ)**


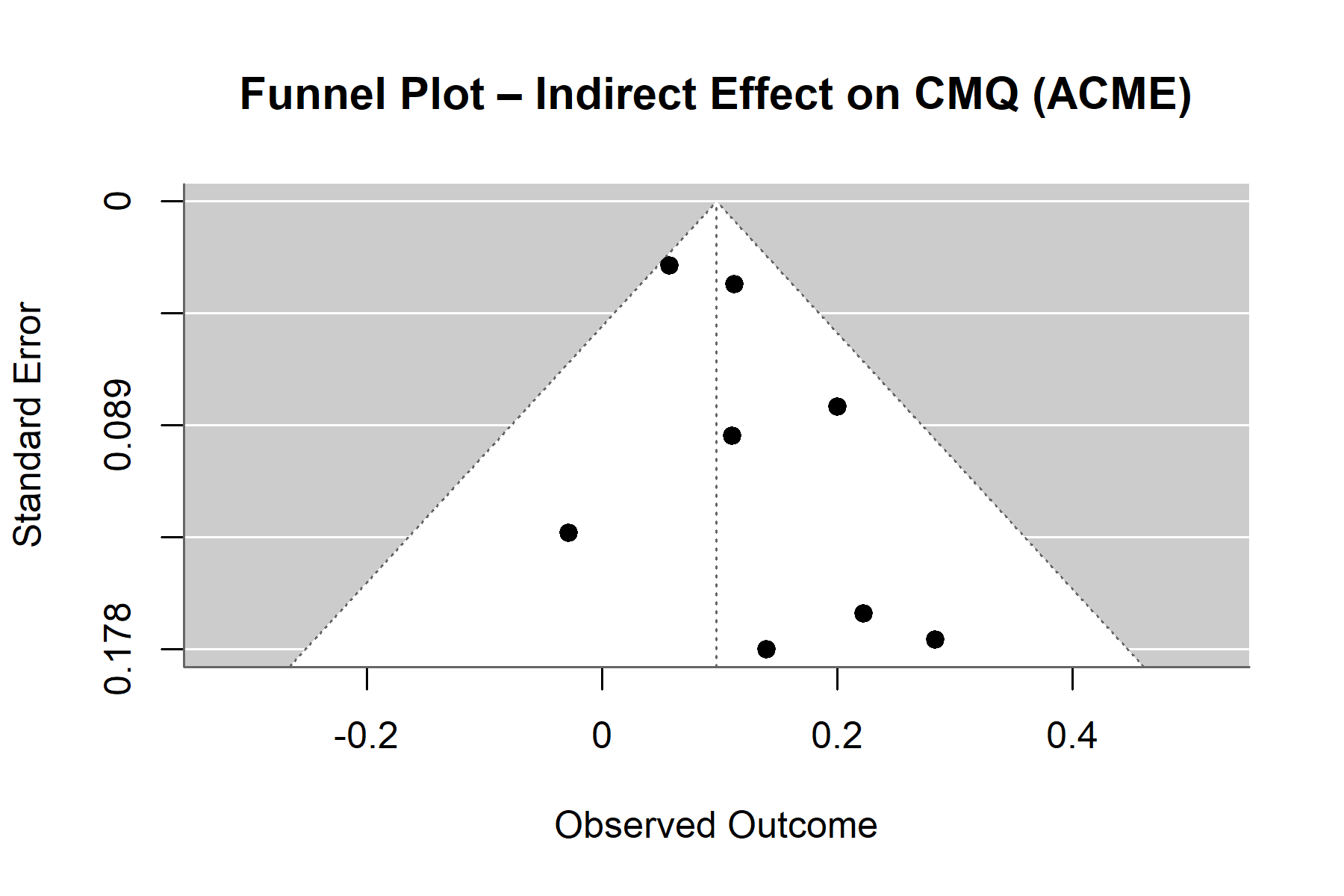
**Figure S2**
*Funnel Plot of Study-Wise Indirect Effects (ACMEs) for Conspiracy Mentality.*
*Note.* Each point represents the indirect effect (ACME) for one study. The vertical line indicates the pooled estimate. The plot provides a visual inspection of potential small-study effects.

**Indirect Effect (RD → Emotions → Specific Beliefs)**


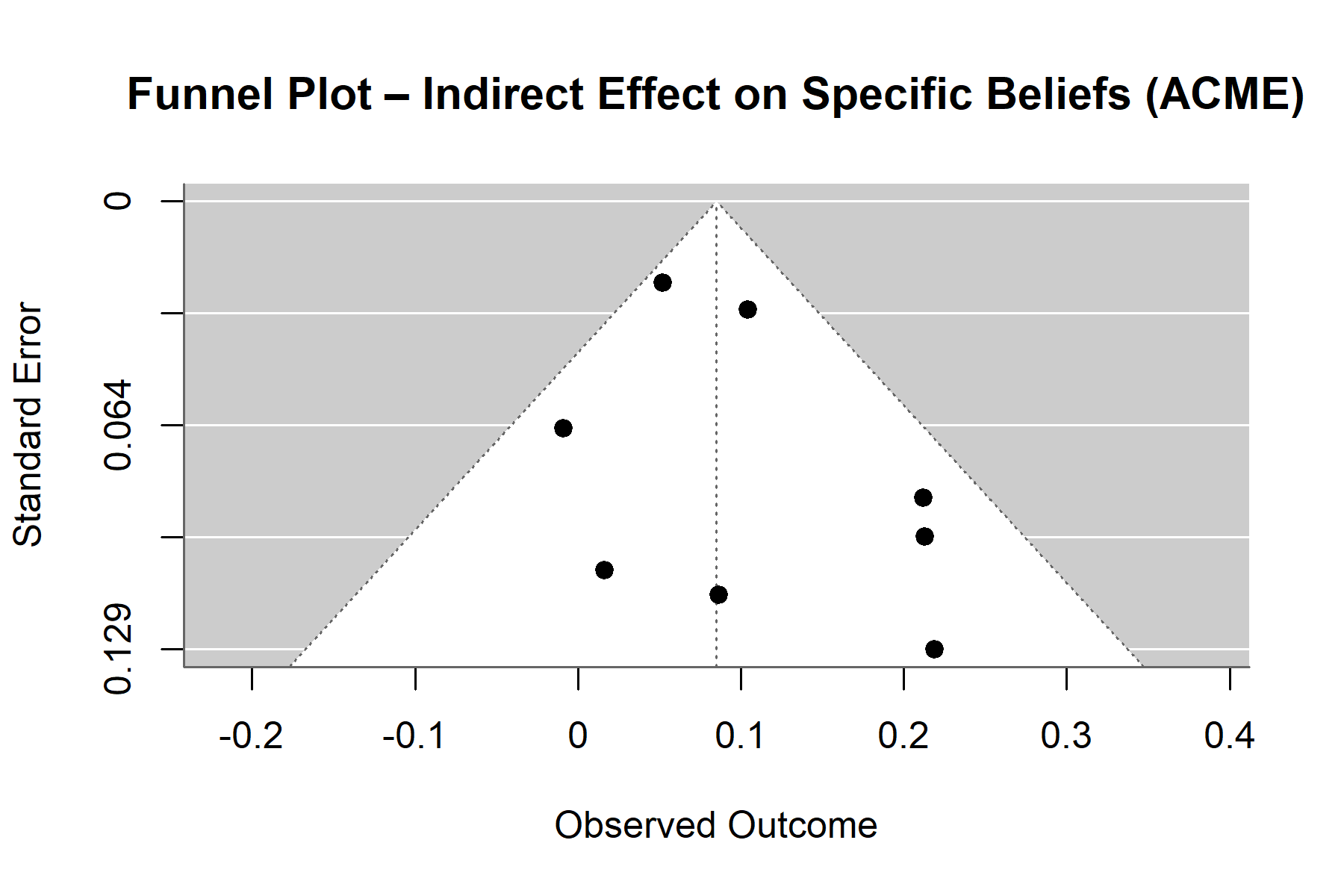
**Figure S3**
*Funnel Plot of Study-Wise Indirect Effects (ACMEs) for Specific Conspiracy Beliefs.*

*Note.* Each point represents the indirect effect (ACME) for one study. The vertical line indicates the pooled estimate. The plot provides a visual inspection of potential asymmetry across studies.

Table S4

*The programmed likelihood of winning for the 10 Keys in both the RD and no-RD after the first 10 trials.*

| Key Number | No-RD | RD Participant | RD Player 2 |  |
| --- | --- | --- | --- | --- |
| 1 | 95% | 95% - 5* trial number | 95% + 5* trial number |  |
| 2 | 85% | 85% - 5* trial number | 85% + 5* trial number |  |
| 3 | 75% | 75% - 5* trial number | 75% + 5* trial number |  |
| 4 | 65% | 65% - 5* trial number | 65% + 5* trial number |  |
| 5 | 55% | 55% - 5* trial number | 55% + 5* trial number |  |
| 6 | 45% | 45% - 5* trial number | 45% + 5* trial number |  |
| 7 | 35% | 35% - 5* trial number | 35% + 5* trial number |  |
| 8 | 25% | 25% - 5* trial number | 25% + 5* trial number |  |
| 9 | 15% | 15% - 5* trial number | 15% + 5* trial number |  |
| 10 | 5% | 5% - 5* trial number | 5% + 5* trial number |  |
| *Note.* RD = Relative deprivation, Key Number = number of coins to bet per round. | | | | |

**Table S5**

*Study-wise mediation results for conspiracy mentality across the eight preregistered studies*

| Study | a (RD → Emotions) | b (Emotions → CMQ) | Indirect Effect (ACME) | Direct Effect (ADE) | Total Effect |
| --- | --- | --- | --- | --- | --- |
| 1 | .678 | .278 | .283 | -.014 | .095 |
| 2 | .764 | .211 | .223 | -.064 | -.023 |
| 3 | .625 | .222 | .200 | -.044 | -.009 |
| 4 | .441 | .161 | .111 | -.106 | -.163 |
| 5 | .791 | .129 | .140 | .014 | .095 |
| 6 | .758 | -.026 | -.029 | .013 | .014 |
| 7 | .168 | .228 | .057 | -.046 | -.065 |
| 8 | .320 | .228 | .112 | -.034 | .022 |

*Note*. a represents the effect of RD on negative emotions; b represents the association between negative emotions and conspiracy mentality controlling for condition. ACME = average causal mediation effect; ADE = average direct effect. Total effects represent the effect of RD on the outcome variable without including the mediator. Estimates are reported separately for each study and are unstandardized.

**Table S6**

*Study-wise mediation results across the eight preregistered studies*

| Study | a (RD → Emotions) | b (Emotions →Specific CB) | Indirect Effect (ACME) | Direct Effect (ADE) | Total Effect |
| --- | --- | --- | --- | --- | --- |
| 1 | .678 | .214 | .218 | -.086 | -.047 |
| 2 | .764 | .201 | .213 | -.076 | .006 |
| 3 | .625 | .235 | .212 | .016 | .133 |
| 4 | .441 | -.013 | -.009 | -.036 | -.078 |
| 5 | .791 | .080 | .086 | .005 | .069 |
| 6 | .758 | .015 | .016 | -.029 | -.052 |
| 7 | .168 | .208 | .052 | -.032 | -.039 |
| 8 | .320 | .211 | .104 | -.044 | -.043 |

*Note.* *a* represents the effect of RD on negative emotions; *b* represents the association between negative emotions and specific conspiracy beliefs controlling for condition. ACME = average causal mediation effect; ADE = average direct effect. Total effects represent the effect of RD on the outcome variable without including the mediator. Estimates are reported separately for each study and are unstandardized.
